# Multi-Scale Correlative Tomography of a Li-Ion Battery Composite Cathode

**DOI:** 10.1038/srep30109

**Published:** 2016-07-26

**Authors:** Riko Moroni, Markus Börner, Lukas Zielke, Melanie Schroeder, Sascha Nowak, Martin Winter, Ingo Manke, Roland Zengerle, Simon Thiele

**Affiliations:** 1 Laboratory for MEMS Applications, IMTEK Department of Microsystems Engineering, University of Freiburg, Georges-Koehler-Allee 103, 79110 Freiburg, Germany; 2 MEET Battery Research Center, Institute of Physical Chemistry, University of Münster, Corrensstraße 46, 48149 Münster, Germany; 3 Helmholtz Centre Berlin, Hahn-Meitner-Platz 1, 14109 Berlin, Germany; 4 Hahn-Schickard, Georges-Koehler-Allee 103, 79110 Freiburg, Germany; 5 FIT, University of Freiburg, Stefan-Meier-Straße 21, 79104 Freiburg, Germany

## Abstract

Focused ion beam/scanning electron microscopy tomography (FIB/SEMt) and synchrotron X-ray tomography (Xt) are used to investigate the same lithium manganese oxide composite cathode at the same specific spot. This correlative approach allows the investigation of three central issues in the tomographic analysis of composite battery electrodes: (i) Validation of state-of-the-art binary active material (AM) segmentation: Although threshold segmentation by standard algorithms leads to very good segmentation results, limited Xt resolution results in an AM underestimation of 6 vol% and severe overestimation of AM connectivity. (ii) Carbon binder domain (CBD) segmentation in Xt data: While threshold segmentation cannot be applied for this purpose, a suitable classification method is introduced. Based on correlative tomography, it allows for reliable ternary segmentation of Xt data into the pore space, CBD, and AM. (iii) Pore space analysis in the micrometer regime: This segmentation technique is applied to an Xt reconstruction with several hundred microns edge length, thus validating the segmentation of pores within the micrometer regime for the first time. The analyzed cathode volume exhibits a bimodal pore size distribution in the ranges between 0–1 μm and 1–12 μm. These ranges can be attributed to different pore formation mechanisms.

Due to high demands for energy storage solutions, it is necessary to further improve the performance of well-established systems, such as lithium-ion batteries^1^,^2^. Therefore, a deeper insight has to be gained into the highly complex electrochemical transport mechanisms in these devices^3^. In general, a commercially available lithium-ion battery consists of a carbon based anode, a lithium metal oxide cathode, and a polyolefin-based separator soaked with an electrolyte located between the anode and cathode^4^. The electrolyte is liquid and usually contains a mixture of linear and cyclic organic carbonates as well as conducting salt^5^,^6^. The composite electrodes consist of at least four material domains: active material (AM), carbon black filled polymer binder domain (CBD), intergranular pore space (PS), and metallic current collector^7^. Lithium storage takes place in the AM, the CBD both electronically connects the AM particles and provides electrical contact between AM and current collector. The electrolyte filled PS enables ion transport within the electrode. The micro- and nanostructure of the respective material domains each have a significant impact on the transport properties of the electrodes^7^,^8^,^9^ and thus are a key to understand ionic and electronic conductivity and their corresponding processes.

Different tomographic techniques are available for materials science to resolve and reconstruct electrode structures. In order to obtain a useful physical analysis of a tomographic reconstruction, it has to be segmented, that is categorized, into its different material domains. This segmentation has an immense impact on both the volume and surface area of the reconstructed material domains, i.e. its morphology. The calculation of transport properties is highly sensitive to this step and therefore relies on a high-quality segmentation^10^. The significance of this step requires that the tomographic method provides both good material contrast and high spatial resolution. For Li-ion battery electrodes, two main techniques meet these requirements: focused ion beam/scanning electron microscopy tomography (FIB/SEMt) and X-ray tomography (Xt)^11^,^12^.

FIB/SEMt is a destructive tomographic approach, which can be used to create reconstructions of all relevant material domains present in composite electrodes^13^,^14^,^15^,^16^. FIB/SEMt provides resolutions of up to 10 nm and allows for different detector systems that can be used in parallel. One of the main drawbacks of this method is its restricted field of view—only sample volumes of up to (40 μm)^3^ can be reconstructed within reasonable time and cost scales^17^.

This volume restriction does not apply to Xt, although spatial resolution and field of view are not independent from each other in this technique. Usually, these parameters are chosen with respect to the median particle size within the component of interest. Operating modes, such as phase contrast imaging, provides the high-contrast data that is needed to perform reliable segmentation^18^, making feasible ternary electrode reconstructions of a few micrometer side length, with resolutions up to 50 nm^19^,^20^. However, due to AM particle sizes of about 20 μm, common lithium battery electrode reconstructions show resolutions of about half a micrometer and side lengths of several hundred micrometers^21^,^22^,^23^,^24^,^25^.

In the case of lithium metal oxide composite cathodes, the combination of a high mass attenuation coefficient of the AM in comparison to the CBD and pore structures with different length scales hinders the ability to image of all relevant material domains in a representative manner. Thus, the common tomographic approach is to image solely the AM and neither the CBD nor the PS^23^,^25^. Recent studies, however, have shown that the CBD morphology, on both the micrometer and nanometer scale, determines the electronic^25^ and ionic^26^ conduction in the composite electrode. Since lithium metal oxides are the most common AM in Li-ion battery cathodes, the reconstruction of all three material domains in these cathodes is of particular interest. For this purpose, a correlative approach, consisting of Xt and FIB/SEMt^27^,^28^,^29^,^30^, was used: both Xt and FIB/SEMt have been performed on the same lithium manganese oxide (LiMn_2_O_4_; LMO) composite cathode at the exact same spot. This allows not only an analysis and validation of state-of-the-art Xt segmentation by a different tomographic technique, but also the extraction of both the LMO and CBD structures on a scale of several hundred microns for the first time.

## Materials and Methods

**Figure 1:**
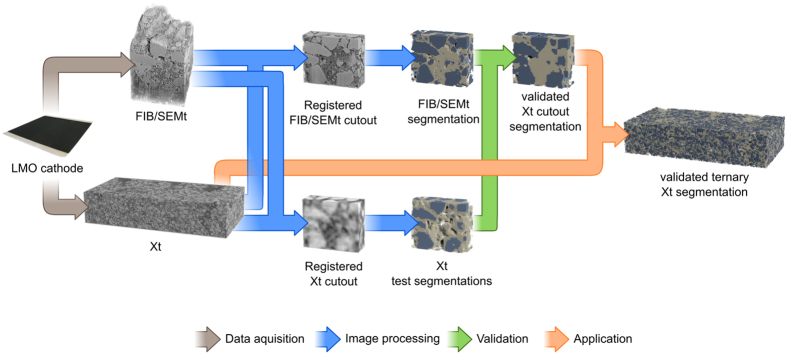
The working principle of correlated tomography. From the left to the right: Sample preparation, Xt and FIB/SEMt data acquisition, data registration, evaluation of the best Xt segmentation on the basis of best agreement with FIB/SEMt segmentation, and application of this method to a large-scale Xt reconstruction.

In order to quantify the quality of common Xt segmentation methods and to evaluate the best approach, this work followed the process depicted in Fig. 1. First, an LMO composite cathode was prepared. This cathode was then imaged by means of FIB/SEMt and Xt. Afterwards, both tomography datasets were registered, i.e. virtually aligned, with each other. The experimental details of the method are described in this section.

### Sample Preparation

The examined composite cathode was prepared using 90 wt% LMO and 10 wt% CBD (7 wt% Super P, 2 wt% carboxymethyl cellulose (CMC), and 1 wt% styrene-butadiene rubber (SBR)). The dry film thickness of the cathode is 121.1 μm (measured with Xt), corresponding to a mass loading of 21.1 mg/cm^2^. This results in a porosity of


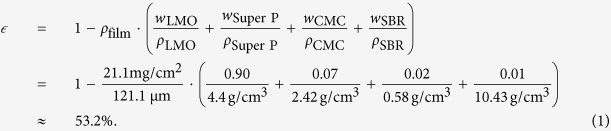


On the basis of this porosity, the volume fractions within the electrode can be calculated to 35.6 vol% LMO and 11.2 vol% CBD.

### X-ray Tomography

The composite cathode was cut and subsequently imaged using synchrotron phase-contrast Xt at BESSY, Helmholtz Centre Berlin, as described elsewhere^31^. The reconstructed dataset consists of voxels with an edge length of 438 nm. Each voxel has an integer gray value between 0 and 255, as a function of the corresponding material properties. For this work, two cutouts of the overall dataset have been used: a large-scale cutout of 500 μm × 110 μm × 250 μm and a small-scale cutout of 28.5 μm × 24.1 μm × 10.5 μm, corresponding to the volume imaged by FIB/SEMt.

### Focused Ion Beam/Scanning Electron Microscopy Tomography

FIB/SEMt was performed, after the Xt, at MEET, as described in another publication^14^, using an Auriga CrossBeam workstation from Zeiss with a field emission gun (Schottky-type). The SEM and the Xt reconstruction were used to navigate over the sample and to search for a spot that showed distinctive features which were observable by both methods, such as large AM grains or electrode cracks. The cross-sections for the tomography were prepared by a FIB milling process, using gallium ions from a high brightness liquid metal ion source. Two separate detectors, a secondary electron/secondary ion (SESI) detector and an in-lens detector, were used for data acquisition, which resulted in a reconstruction of 31.3 μm × 34.5 μm × 16.8 μm and a voxel size of (33.6 nm)^3^.

### Preparation of Ground Truth

Although the volume imaged by FIB/SEMt is not a representative volume element for the investigated composite cathode and thus not suitable for the direct calculation of transport properties, it contains sufficient statistical information to be used as ground truth for the evaluation of Xt segmentation. For this purpose, the FIB/SEMt dataset was registered with the Xt reconstruction by means of maximization of mutual information using MATLAB^32^. Subsequently, the edges of the registered FIB/SEMt reconstruction were virtually cut again, in order to obtain a cuboid of 28.5 μm × 24.1 μm × 10.5 μm size. This cuboid was then segmented into the AM, CBD, and PS domains and afterwards coarsened to Xt resolution according to the majority rule^33^. Finally, the features of each material domain with a diameter below the resolution limit of the Xt data, assumed to be two voxel sizes, were excluded from further analysis. This reduces the influence of features which are not detectable as a matter of principle on the evaluation.

### Segmentation Evaluation Measure

In order to reliably compare different segmentation approaches of the physically identical data, a defined measure of ‘similarity’ has to be applied. In this work, Cohen’s *κ*^34^ is used, since it is easily implementable, widely spread, and enables comparison of multi-domain segmentations^35^. It is defined as


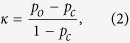


where *p*_*o*_ is the observed agreement and *p*_*c*_ is the agreement that is expected by chance. A common interpretation of *κ* assigns values below 0 as poor, values between 0 and 0.2 as slight, values between 0.2 and 0.4 as fair, values between 0.4 and 0.6 as moderate, values between 0.6 and 0.8 as substantial, and values between 0.8 and 1 as an almost perfect agreement^36^.

### Estimation of Pore and Grain Size Distributions

Calculating the pore and grain sizes, on the basis of the respective segmentations and their comparison, allows for further evaluation of the different approaches to segmentation. In contrast to the objective, but rather abstract, *κ* value, pore and grain sizes are real world quantities and represent the morphological features of the reconstructed electrode volumes. Within this work, two distinct representations of pore and grain size distributions are shown; the spatial size distribution of the segmented data and the corresponding density function. The latter was determined by using a quartic kernel density estimation, with a bandwidth of one voxel side length, i.e. the estimated accuracy of the calculated sizes. Both representations are given in order to allow intuitive comparison between segmentations.

## Results and Discussion

In this section, an evaluation of state-of-the-art binary battery electrode Xt segmentation is presented in terms of AM domain and non-AM domain. This evaluation compares the domains of the Xt and FIB/SEMt datasets, as described above. Secondly, a complete ternary segmentation of the Xt dataset is introduced, consisting of the AM, CBD, and PS, and validated against the FIB/SEMt ground truth. Finally, this segmentation is applied to a large cutout of the Xt dataset, in order to understand the ternary microstructure of the LMO composite cathode investigated in this work.

### Binary Xt Segmentation

State-of-the-art AM segmentation is usually performed by simple gray value thresholding, according to visual judgement with respect to a histogram of the image data^20^,^23^,^25^. However, until now, no analytical validation of this method is available in literature. The application of the correlative tomography method introduced here provides an approach to achieve this goal.

**Figure 2:**
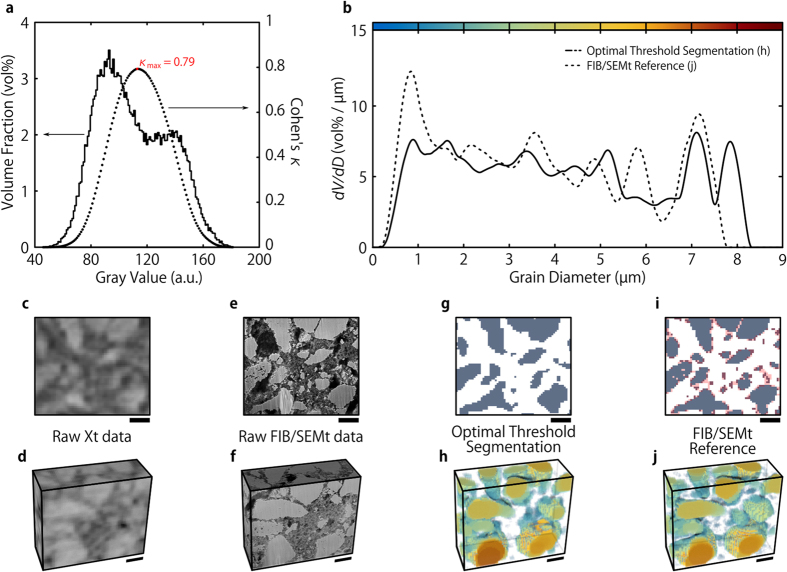
The estimation of the quality of binary Xt threshold segmentation by means of Cohen’s *κ* and comparison of the AM structure. (**a**) The histogram of the Xt data shown in c and the quality of AM segmentation by means of Cohen’s *κ* for each possible threshold value. (**b**) The grain size distributions of Xt and FIB/SEMt AM calculated from e and g, respectively. (**c**) A 2D Xt image of the correlatively imaged volume. (**d**) A 3D representation of the correlatively imaged volume as seen with Xt. (**e**) A FIB/SEMt image corresponding to (**c**). (**f**) A 3D representation of the correlatively imaged volume as seen with FIB/SEMt. (**g**) The best AM Xt segmentation by threshold application as determined in (**a**), applied to the data shown in (**c**). AM is shown in gray. (**h**) The corresponding spatial grain size distribution in 3D. (**i**) A 2D image of the coarsened FIB/SEMt segmentation that was used as reference in (**a**). Features shaded in red were considered too small for detection by Xt and therefore excluded from comparison. (**j**) The spatial grain size distribution of the FIB/SEMt AM segmentation in 3D. The scale bars in (**c–j**) correspond to 5 μm.

The quality of this LMO segmentation method was quantified by calculating Cohen’s *κ* for every possible gray value threshold (Fig. 2). Gray values above the threshold were assigned to LMO, while voxels with gray values below the threshold were defined as non-LMO. Maximum similarity (*κ*_max_ = 0.79, i.e. close to perfect agreement) was found for a threshold value of 113 (Fig. 2a,g,h). This threshold is very close to values obtained by standard gray level thresholding algorithms, such as Otsu’s method (110)^37^ or entropy based methods (113)^38^.

Visual comparing the best LMO segmentation to the FIB/SEMt data reveals that the main source of error stems from the low material contrast of small features: AM grains of a few voxel lengths in diameter often appear too dark in Xt data to be assigned correctly and many spatially proximate LMO grains appear connected in Xt although being separated. While the coarsened FIB/SEMt segmentation exhibits 81 separate LMO grains, Xt segmentation only shows 17. This significant overestimation of connectivity results in an underestimation of both the LMO volume and surface area by 6% and 25%, respectively. These observations are repeated in the visualization of the local grain size^39^ of the Xt segmentation (Fig. 2h): Although overall in very good agreement with the FIB/SEMt reference (Fig. 2j), small grains, shaded solely in blue, appear less often in the Xt dataset. Hence, the grain size distribution (GSD) of the Xt data (Fig. 2b), although close to the reference, underestimates the amount of small grain diameters and is slightly shifted towards larger grain diameters.

### Complete Ternary Xt Segmentation

State-of-the-art segmentation in lithium metal oxide composite cathodes does not provide good CBD classification in Xt images, due to low material contrast and, as a result, high levels of uncertainty. The correlative tomography approach applied here not only allows for the quality of the AM segmentation to be quantified, but also can be used to realize a reliable ternary segmentation method for the Xt dataset.

**Figure 3:**
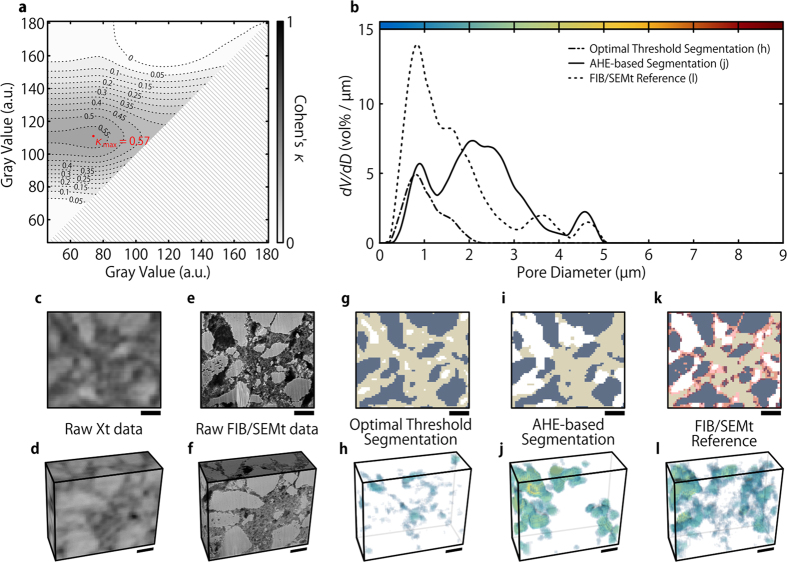
The estimation of the quality of two different ternary Xt segmentation methods, by means of Cohen’s *κ* and comparison of the pore structure. (**a**) Cohen’s *κ* for each possible combination of AM and CBD threshold values. (**b**) The pore size distributions of Xt and FIB/SEMt, calculated from (**h,j,l**), respectively. (**c**) A 2D Xt image of the correlatively imaged volume. (**d**) A 3D representation of the correlatively imaged volume as seen with Xt. (**e**) A FIB/SEMt image corresponding to (**c**). (**f**) A 3D representation of the correlatively imaged volume as seen with FIB/SEMt. (**g**) The best ternary Xt segmentation as determined in a, visualized by application to the 2D Xt image in (**c**). The AM and CBD are gray and light beige, respectively. (**h**) The corresponding spatial pore size distribution in 3D. (**i**) The same Xt data as used in (**g**), segmented by the AHE-based segmentation described within this work. (**j**) The corresponding spatial pore size distribution in 3D. (**k**) A 2D image of the FIB/SEMt segmentation that was used as reference. Features shaded in red were considered too small for detection by Xt and therefore excluded from comparison. (**l**) The spatial pore size distribution of the FIB/SEMt AM segmentation in 3D. The scale bars in (**c–l**) correspond to 5 μm.

For this purpose, the procedure described in subsection 3.1 was adapted for three material domains, by applying two gray level thresholds to the Xt data. In order to find the most suitable values, every possible threshold pair was compared to the ground truth by means of Cohen’s κ (Fig. 3a), under the assumption that the gray level of CBD voxels is smaller than the gray level of AM voxels. The optimum thresholds lead to a gray value classification of 0 to 73 as PS, 74 to 110 as CBD and 111 to 255 as AM. The slight change in the optimal AM threshold value is caused by the increase of the presumed material domains from two to three. Cohen’s κ_max_ is calculated to be 0.57, i.e. moderate agreement, and visual comparison to the ground truth shows that this segmentation method is by no means sufficient (Fig. 3a,b,g,h). However, the comparison provides a means to determine and analyze areas of low similarity between the Xt data and the ground truth in order to improve the classification.

**Figure 4:**
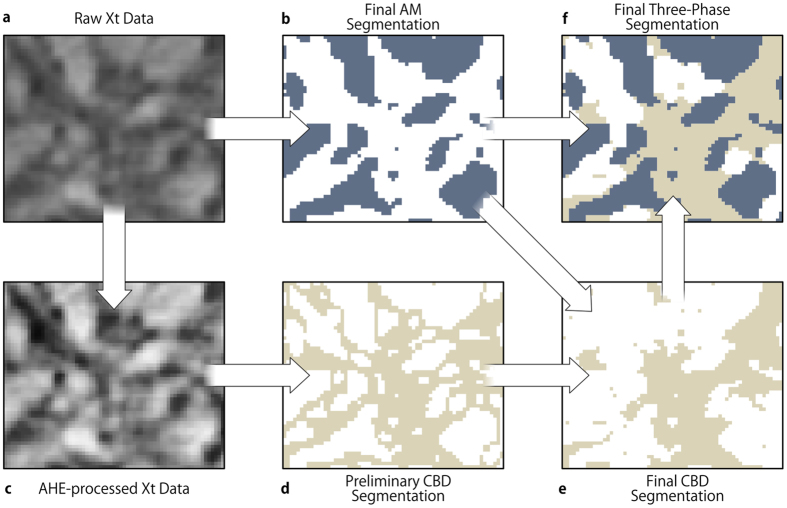
The steps of the AHE-based segmentation approach used in this work. AM and CBD are gray and light beige, respectively. (**a**) A 2D Xt image of the correlatively imaged volume. (**b**) The AM segmentation gained by threshold application. (**c**) AHE processed version of a. (**d**) The CBD segmentation achieved by application of a lower and upper gray value threshold. (**e**) The final CBD segmentation after performing a morphological interpolation of features smaller than two voxels (resolution limit) in (**c**). The interpolation is performed with respect to the AM segmentation. (**f**) Final segmented image containing all three composite electrode material domains.

This analysis revealed that the local material contrast relies, to a certain degree, on the material distribution within its neighborhood. This leads to the segmentation process described in Fig. 4: For the AM, the precise segmentation as described in section 3.1 was utilized (Fig. 4b). However, in order to achieve an accurate CBD/PS segmentation, the segmentation method had to be optimized, resulting in three steps: (i) Application of an adaptive histogram equalization (AHE)^40^ with respect to the typical AM grain distance (Fig. 4c). (ii) Application of an upper and lower gray level threshold (Fig. 4d). The same upper threshold in the AM segmentation is used. (iii) Removal of features smaller than the assumed resolution limit (two voxel sizes) as well as morphological interpolation of the CBD below this limit (Fig. 4e). In the last step it is assumed that the CBD interconnects the AM particles and thin, freestanding, or other peculiar structures are unfavored and thus negligible. In terms of image processing, this corresponds to a morphological opening of the CBD and a subsequent closing of the set union of CBD and AM. The compounded structure of this processed CBD and the AM classification forms the final AHE-based segmentation, which leads to a significantly improved similarity to the ground truth with *κ*_max_ = 0.66 (Fig. 3i,j).

To assess the morphological similarity between ground truth and Xt segmentations, the respective local pore sizes and the corresponding pore size distributions (Fig. 3b,h,j,l) were calculated. As expected from visual judgement, the best threshold segmentation shows little to no similarity to the ground truth. The pore space is composited of small pores (shown as blue voxels in Fig. 3h) that are uniformly distributed within the CBD. No pores larger than two microns occur, due to the massive overestimation of CBD volume. In contrast, the AHE segmentation works very well when above the assumed resolution limit of two voxel lengths, i.e. above 1 μm. Despite an overestimation of the pore diameters by about one micron, the AHE-based pore size distribution (PSD) matches with the ground truth PSD. The overestimation originates in the resolution limit, since small CBD features that protrude into the pores in the reference are undetectable by Xt, thus resulting in larger pore sizes. The visual juxtaposition of the local pore diameter of the AHE-based segmentation and the FIB/SEMt reference (Fig. 3j,l) supports this observation: While the small pores (blue) seen in the reference are not detected by Xt, almost all pores above the resolution limit (green to yellow) are identified correctly. This substantial agreement between ground truth and AHE-based segmentation allows for a reasonable transfer of this segmentation method to be applied from the correlatively imaged volume to a larger Xt dataset, representative of the three domains, which is described in the next subsection.

### Ternary Segmentation of a Representative Xt Dataset

Until now, acquiring a ternary segmentation of lithium metal oxide composite electrode reconstructions into the AM, CBD, and PS, within an affordable time and cost, could only be achieved with techniques that allow imaging of volumes with few micrometers side length. Due to AM particles and pores sizes consisting of several micrometers in diameter, imaging a representative fraction of the composite cathode is not possible with FIB/SEMt due to the limited field of view. The AHE-based segmentation, deduced from the correlative tomography approach, allows performing a ternary segmentation of an Xt reconstruction that provides representative information in the range of several hundred micrometers.

**Figure 5:**
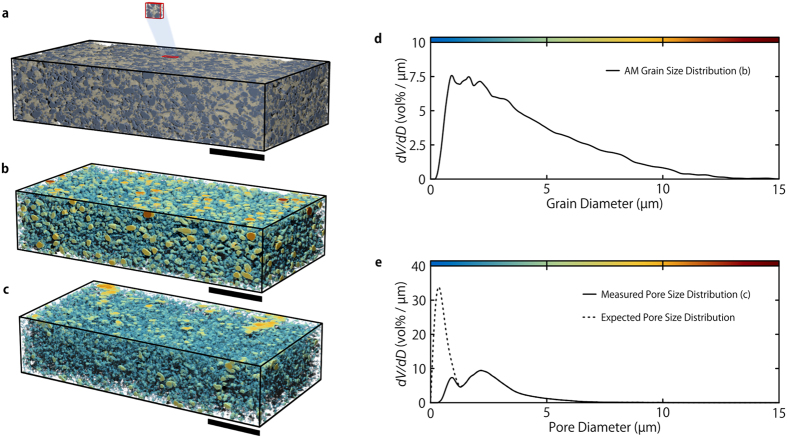
A 500 μm × 110 μm × 250 μm ternary Xt segmentation of the investigated battery electrode and its corresponding size distributions. (**a**) A 3D representation of the relevant electrode subvolume. The correlatively imaged volume is highlighted in order to show a size comparison. The AM and CBD are gray and light beige, respectively; the black bar corresponds to a length of 100 μm. (**b**) The local grain size distribution within the volume shown in (**a**). (**c**) The corresponding local pore size distribution. (**d**) The estimated grain size distribution as calculated from (**b**). (**e**) The estimated pore size distribution that corresponds to (**c**) (solid line). Since the majority of the pore volume lies below the resolution limit of the Xt, the dashed line indicates the expected progression of the PSD towards smaller diameters.

For this purpose, the segmentation approach discussed in subsection 3.2 was applied to a 500 μm × 110 μm × 250 μm large subvolume of the X-ray reconstruction (Fig. 5a and Figure S1). This section was chosen with respect to the homogeneity of the electrode, i.e. no larger cracks or pores appear within this subvolume. After the segmentation has been applied, the measured porosity is 24.8 vol% (53.3 vol%), the AM fraction is 38.3 vol% (35.6 vol%), and the CBD volume fraction is 36.9 vol% (11.2 vol%). The values denoted in brackets are calculated on the basis of the measured electrode dry film thickness (Section 2.1).

The deviation between these figures is apparent; although the agreement between expected and measured AM fraction is adequate, the Xt segmentation overestimates the volume of active material by 7.6%. The segmented volume fractions of CBD and PS deviate even more from the expected values. However, both deviations were expected. The slight overestimation of AM volume fraction is within an acceptable error range^25^ and additionally supports the validity of the segmentation. Since the investigated electrode volume is free from cracks or other defects, the amount of AM within the subvolume should be larger than the electrode average. This also applies to CBD segmentation. Nevertheless, the main reason for the deviation between CBD and pore volume fractions lies within the available resolution. Pores smaller than one micrometer are simply not detected and thus interpreted as CBD, and since the segmentation does not treat fuzziness any differently, segmented voxels are either considered as massive CBD or PS. This interpretation is obviously wrong, as shown by the FIB/SEMt—voxels segmented as CBD exhibit an inherent porosity. The CBD’s average porosity can be calculated by taking the known value of the electrode as a basis, resulting in 77.0% mean CBD porosity. Naturally, the values for this figure currently available in literature were obtained using FIB/SEM-based techniques and thus are not directly comparable to Xt. The comparison between volume fractions of Xt and non-coarsened FIB/SEMt segmentation show that approximately 18.5% of the pore volume seen by FIB/SEMt is not detected by Xt, due to the influence of resolution and measuring method. This leads to an estimated CBD porosity of 62.7% for the resolution range of the FIB/SEMt, which resembles values reported in literature^16^,^41^. The strong dependence of measurable porosity and tomographic resolution is shown in Table S1.

The Xt segmentation allows for analysis of both grain and pore sizes within the investigated electrode volume (Fig. 5b–d). The AM GSD exhibits a single peak and ranges from 0 μm to 15 μm with an expected value of 4.0 μm. It corresponds roughly to a log-normal distribution with a location 1.22 [μm] and a scale of 0.86 (R^2^ = 0.97). The homogenous spatial distribution of AM grain sizes throughout the volume (Fig. 5b) and the ratio of the maximum grain diameter to minimal volume side length indicate that the chosen electrode volume is indeed representative.

In contrast to the GSD, the PSD shows two distinct peaks at 0.9 μm and 2.2 μm and thus cannot be satisfactorily described with a common probability distribution. Its expected value lies at 2.6 μm, where the largest pore has a diameter of about 12 μm. The bimodal shape of the PSD is of particular interest. It becomes even more apparent when considering the sub-resolution porosity, which makes up 53.4% of the overall pore volume. The radical change in the PSD, by incorporation of these pores, is indicated by the dotted line in Fig. 5d. The bimodality can be attributed to different effects during electrode preparation: (i) Pores larger than 1 μm originate from the backbone structure built by the AM. Although the AM is interconnected by the CBD, a substantial amount of cavities between the particles remain. The morphology of the AM particles thus has an influence on the size and shape of the pores, as well as the overall porosity of the composite cathode^42^. The occasional entrapment of small gas bubbles during electrode preparation also contributes to pores in the micrometer regime. (ii) Small pores with diameter below 1 μm stem from the three-dimensional structure of carbon black and binder^41^. These pores represent the largest fraction of the PS (see Fig. 5e, dotted line).

The bimodality of the PSD is intrinsically associated with the electrochemical performance of Li-ion battery composite cathodes, in terms of good electrode kinetics and complete utilization of AM. While the microporous structure of the CBD creates an electrically conductive network within the electrode, the larger pores are necessary to facilitate a complete penetration of the electrolyte into the microstructure of the electrode. A dense structure of micropores would inhibit the electrolyte wetting, therefore inhibiting the ion transport, leading to an incomplete utilization of active material and ultimately an insufficient energy density.

The general morphology of the PS is affected by numerous parameters, like the material selection, material composition, drying kinetics, and mechanical stress during the preparation process^43^,^44^,^45^,^46^. Although the influence these parameters make is of great importance on the properties of the electron and ion conducting networks within the composite electrode, the systematic investigation into relations between the process parameters and microstructural properties, using correlated tomography, is far beyond the scope of this work.

## Conclusion

The influence of the CBD on the transport properties of Li-ion battery composite cathodes can only be understood by the application of multiple tomographic approaches. Until now, the CBD morphology on the micrometer range has only been reconstructed by means of multi-scale modeling. This work presents a novel and much more accurate method of analysis on the basis of correlative FIB/SEMt and Xt.

Furthermore, correlative tomography has validated the segmentation approaches for the AM domain. For the first time, standard threshold approaches were confirmed to be accurate for AM segmentation. Since thresholding algorithms used on high-contrast datasets are shown to be reliable, their usage is strongly advised over visual judgement, due to the inherent arbitrariness visual judgement entails.

The correlative approach also allowed for a segmentation of an Xt composite cathode reconstruction, with several hundred micrometer side length into all relevant material domains. Threshold segmentation is obviously not applicable for this purpose, however, it was used to realize a segmentation method that makes the accurate identification of pores larger than 1 μm feasible. The application of this segmentation revealed two major pore size regimes in the reconstructed composite cathode, which is the result of different pore formation mechanisms. The PS reconstruction demonstrates that correlative tomography is a suitable tool to investigate the interplay between the preparation process parameters and pore morphology in lithium metal oxide composite cathodes.

While the basis for PS analysis in the micrometer regime has been established in this work, nanometer pore morphology still has to be incorporated by spatio-statistical modeling in order to assess and tune transport properties within these cathodes. Interaction and influence of both pore size regimes will be part of future investigations.

Due to the fundamental improvement in image segmentation by application of correlated tomography, it is a key technique to the investigation and understanding of materials with hierarchical microstructure.

## Additional Information

**How to cite this article**: Moroni, R. *et al.* Multi-Scale Correlative Tomography of a Li-Ion Battery Composite Cathode. *Sci. Rep.* **6**, 30109; doi: 10.1038/srep30109 (2016).

## Supplementary Material

Supplementary Information
