# Supplementary material for: Multi-Scale Correlative Tomography of a Li-Ion Battery Composite Cathode
Source: Sci Rep. 2016 Jul 26;6:30109. doi: 10.1038/srep30109 (PMC4960488; doi:10.1038/srep30109)

Multi-Scale Correlative Tomography of a Li-Ion Battery Composite Cathode

Riko Moroni^1^, Markus Börner^2^, Lukas Zielke^1^, Melanie Schroeder^2^, Sascha Nowak^2^, Martin Winter^2^, Ingo Manke^3^, Roland Zengerle^1,4^, Simon Thiele^1,5^,*

^1^ Laboratory for MEMS Applications, IMTEK Department of Microsystems Engineering, University of Freiburg, Georges-Koehler-Allee 103, 79110 Freiburg, Germany

^2^ MEET Battery Research Center, Institute of Physical Chemistry, University of Münster, Corrensstraße 46, 48149 Münster, Germany

^3^ Helmholtz Centre Berlin, Hahn-Meitner-Platz 1, 14109 Berlin, Germany

^4^ Hahn-Schickard, Georges-Koehler-Allee 103, 79110 Freiburg, Germany

^5^ FIT, University of Freiburg, Stefan-Meier-Straße 21, 79104 Freiburg, Germany

* simon.thiele@imtek.uni-freiburg.de

**Supplementary Information**

| **Porosity [%]** | **Pixel Size [nm]** | **Method** | **Source** |
| --- | --- | --- | --- |
| 47 | 2.5 | FIB/SEM cross-section | Zielke et al. (2014)^[25]^ |
| 57.6 | 3 | FIB/SEM tomography | Vierrath et al. (2015)^[38]^ |
| 62.7 | 33.6 | FIB/SEM tomography | This work |
| 63 | 67 | FIB/SEM cross-section | Stephenson et al. (2011)^[16]^ |
| 77.0 | 438 | X-ray tomography | This work |

Table S1. Porosities in Li-ion battery composite cathodes, as measured with different imaging methods and resolutions.

**Figure S1.** The median (a), frontal (b), and transverse plane of the large-scale Xt reconstruction shown in Figure 5 as both raw (left, top, left, respectively) and segmented images. (d) A view of the raw images in a–c in 3D space. (e) The corresponding 3D representation of the segmented images. The AM, CBD and PS are gray, light beige, and white, respectively; the black bar corresponds to a length of 100 µm.


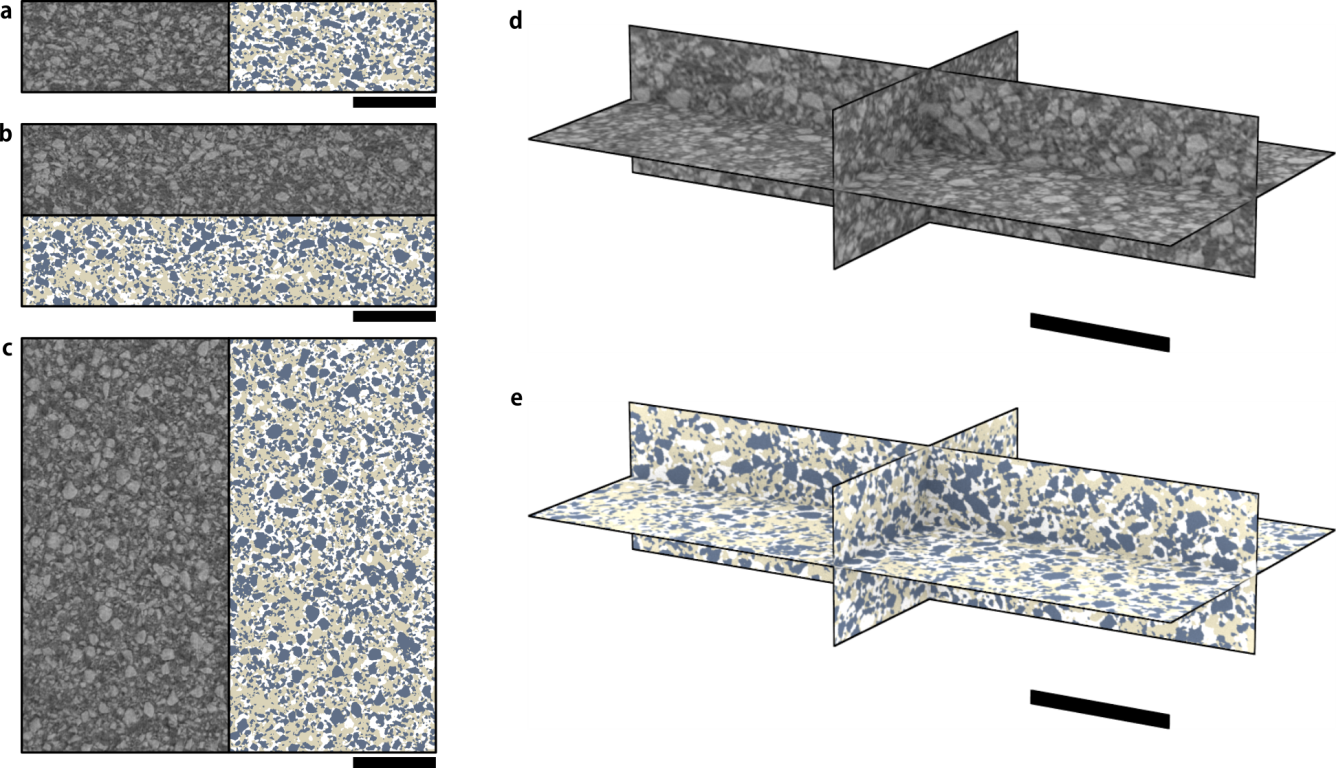

Supplement: Supplementary Information [file srep30109-s1.doc]
